# Editorial: Creative organization development through leadership

**DOI:** 10.3389/fpsyg.2025.1701897

**Published:** 2025-09-30

**Authors:** Osman Titrek, Carlos Francisco de Sousa Reis, Pavel Jurs, Gozde Sezen-Gultekin, Pablo Garcia Sempere

**Affiliations:** ^1^Faculty of Education, Sakarya University, Sakarya, Türkiye; ^2^Faculty of Psychology and Educational Sciences, University of Coimbra, Coimbra, Portugal; ^3^Centre of Pedagogy and Social Work, Riga Technical University Liepaja Academy, Liepaja, Latvia; ^4^Faculty of Education Sciences, University of Granada, Granada, Spain

**Keywords:** creativity, leadership, emotions, leadership types, organizational behavior

## Introduction

The influence of leadership has affected human history for thousands of years, although systematic research on leadership only started in the 20th century. Leadership studies, which produced trait theory, explored behavioral and contingency approaches, with the perspective toward leadership mostly being shaped by these theories until the 1980s. However, some scientists began to question the usefulness of the concept of leadership and thought that leadership studies were limited in their approaches (Hoy and Miskel, 2010). Based on modern theories, leadership is now not confined in definition to the exercise of managerial authority; it can also occur when formal leaders engage in informal behaviors beyond their official responsibilities. For example, Sparrowe and Liden (2005) discovered that, through informal activities, such as a luncheon or a social event, leaders may share valuable contacts in their informal networks by introducing them to their colleagues. For this reason, leadership now refers to a social influence process in which leaders attempt to motivate and enable others to contribute toward achieving collective goals (Bass, 1985, 1990, 2005; Yukl, 2002).

In this context, it is clear that leadership plays an important role in an organization's development and sustainability and that educational organizations need creative leaders. For this reason, this special issue aims to address creativity and leadership in education. Thereby, this issue will draw attention to the contemporary term “creative leadership” and contribute to educational fields by means of exploring the relationship between creativity and leadership. Creativity is developed by guiding rather than being taught. For this, environments that allow rich and diverse experiences should be designed (Vural et al., 2024) and leadership affects lots of different issues in organizations, such as emotions (Goleman, 1998; Goleman et al., 2002, 2005; Batool, 2013; George, 2000; Ginsberg, 2020). In order to build and sustain an organizational culture dominated by creativity, innovation, and digitalization, organizations must effectively identify and mobilize the creative resources of their members. When organizational members experience a work environment that restricts or fails to encourage individual creative expression, a gap may exist between the level of individual creative potential and the actual amount of individual creativity practiced within the organization. Leadership development may represent one important key for unlocking this idle creative potential and enhancing overall organizational effectiveness (Houghton and DiLiello, 2010). Moreover, the ethical behavior of leaders and atmosphere of an organization allows employees to maintain positive emotions (Xiu and Zhao, 2016) and helps leaders and employees create a more creative atmosphere in organizations. So, organizations should focus on leadership to develop creativity via all dimensions and on all levels of the organization. In order to achieve this, reshaping organizational psychology and culture accordingly can be stated as one of the main missions of organizational leadership. Trust is the key factor for leadership (Titrek, 2016) to develop a positive organizational psychology and climate in organizations.

Creativity was initially considered an innate ability that existed primarily in artistic fields. However, it later began to be regarded as a skill that could be developed. As organizational life accelerated, it became apparent that organizations that stood out possessed creative employees. Consequently, developing this skill became important for both employees and those managing the organization. It is also understood that as those managing organizations increase their individual skills, such as creativity, they become more effective as leaders. Recent studies have proven that creativity is necessary for both individuals and organizations. Studies have also shown that leadership is instrumental in encouraging creativity and innovation. Creativity requires an organizational environment where employees feel safe to express new ideas and take risks. In this context, it is clear that creative leadership plays an important role in an organization's development and sustainability. This issue will draw attention to the contemporary term “creative leadership” and contribute to educational fields by means of exploring the relationship between creativity and leadership. Vural et al., discussed how creativity is developed through guidance rather than teaching. For this, environments that allow rich and diverse experiences should be designed. In order to build and sustain an organizational culture dominated by creativity and innovation, organizations must effectively identify and mobilize the creative resources of their members. When organizational members experience a work environment that restricts or fails to encourage individual creative expression, a gap may exist between the level of individual creative potential and the actual amount of individual creativity practiced and developed within the organization via leadership. Houghton and DiLiello explain that leadership development may act to unlock idle creative potential and enhance overall organizational effectiveness. So, organizations should focus on leadership to develop creativity via all dimensions and at all levels of the organization.

We believe that this issue will contribute to the Organizational Psychology Specialty Section by addressing the role of critical concepts in the workplace. Contributions are expected to examine the relationship between different aspects of creative leadership against the backdrop of organizational life, behavior, and psychology. Moreover, this special issue will also reveal the effects of creative organizational culture, which is one of the most fundamental issues in terms of organizational psychology, and the factors affecting this culture in detail. In addition, this special issue aims to reveal how creative organizational culture is changing organizational behavior and psychology as well as organizational structure in today's rapidly changing world. The creative organizational leaders who will produce this change, their characteristics, and the new skills they need to acquire can be stated as another important purpose of this special issue. Leadership today is being redefined—not only as a formal function or title but as an enabler of innovation, adaptability, and ethical transformation in organizations navigating volatile and complex environments. The Research Topic ‘*Creative organization development through leadership*' was launched to investigate these shifting contours and to foster a dialogue around how leadership practices drive sustainable and inclusive development across diverse contexts.

This special issue has attracted attention from different academics all over the world. The high levels of diversity and originality in the contributed studies is a clear indication that it is a significant issue. We have received high-quality submissions that multidimensionally analyze the relationship between emotion, leadership, and its effects on organizational culture, psychology, and education systems, with the aim of thoroughly revealing the subject by accepting academically effective studies. Moreover, I would like to thank the valuable academicians from Türkiye, Brasil, China, Cyprus, Congo, Korea, Spain, Thailand, and New Zealand and the academicians and researchers from many different countries who provided editorial support for this special issue. The 25 articles accepted for this special issue discuss leadership, leadership type, and related topics of leadership in organization. In detail, the studies discuss leadership types (five papers), digital and transformational leadership (six papers), innovation (five papers), creativity (two papers), emotions (two papers), ethical leadership (two papers), leadership competencies (two papers), gender issues (two papers), organizational development and behavior (all papers) and other related topics such as psychological safety, physical capacity, organizational security, organizational trust, school effectiveness, job satisfaction, team reflexivity, diversity, organizational healing, leader tolerance, identification, agility, and self-esteem. When all Research Topics are examined, it is seen that the focus is on the effects of leadership types on organizational behavior and organizational psychology.

Aquino et al. classified interpersonal and intrapersonal leadership competencies into five competency areas and examined the interpersonal and intrapersonal influences in detail. They demonstrated that effective leadership operates at both the individual and interpersonal levels. They stated that leaders significantly influence their internal processes, relationships, and interactions. Resilient leaders stabilize teams during crises; foster a commitment to continuous learning, innovation, collaboration, and knowledge sharing with strong communication skills; and strengthen relationships with customers, subordinates, and stakeholders. Empathetic leadership creates healthier work environments by reducing stress and conflict, fostering motivation and commitment, and fostering team transformation and adaptability. Therefore, these leadership competencies are crucial for maintaining a competitive advantage.

Yuyi et al. also investigated the impact of platform leadership on employee innovation behavior. This study demonstrated the positive impact of leadership on organizational learning, knowledge sharing, peer support, and psychological empowerment and demonstrated the contributions of platform leadership to organizational development. These factors significantly increase employees' innovation behavior, highlighting the importance of developing a learning-oriented culture, encouraging knowledge sharing, and psychologically empowering employees, suggesting applicable insights for leadership policies.

Qasim and Laghari examined the concepts of ethical leadership, creativity, and psychological safety using an ethical climate as a moderator. Their study findings indicated that ethical leadership is a significant direct and indirect factor influencing creativity through a sense of belonging and psychological safety, and that the moderating role of an ethical climate is also significant. The study found that ethical leadership is a strong predictor of sense of belonging and psychological safety, which contribute to employees' creativity. Moreover, Zhu et al. also examined the ethical behavior of leaders and investigated the causal model of radical innovation in China, examining the mediating effect of leader identity and the moderating effect of promotion orientation. They found that leader ethical behavior positively influenced both leader identity and radical innovation. Furthermore, leader identity mediated the relationship between leader ethical behavior and radical innovation. They concluded that promotion orientation strengthened the effect of leader ethical behavior and leader identity on radical innovation.

Some researchers have conducted research examining leadership styles and the emotional dimension of the organization. Bai and He investigated the “double-edged sword” effect of shared leadership on employee voice behavior. On the one hand, their study shows that shared leadership as an empowerment mechanism positively influences employees' voice behavior through empowerment associated with perception of organizational status. On the other hand, shared leadership as an exhaustion mechanism suggests that it negatively impacts employee voice behavior through the service pathway characterized by emotional exhaustion. It is suggested that employees' expectations of empowerment play a critical role in triggering these opposing mechanisms, and that high employee expectations of empowerment increase the positive effects of the empowerment mechanism while reducing the negative impact of the depletion mechanism. Ma et al. examined the roles of organizational behavior, belonging, and organization-based self-esteem in inclusive leadership and subordinates' career goals. This research provides a deeper understanding of the relationships between leadership styles and employees' job satisfaction, identifying a positive relationship between inclusive leadership and career goals. Furthermore, they found that organizational belonging also mediated the relationship between inclusive leadership and career goals. They concluded that organizational centrality and self-esteem moderated the relationship between inclusive leadership and career goals both directly and indirectly through belonging. Liu C.-E. et al. reported that an error-avoidance climate positively influences poor leadership, and that moral disengagement and ego depletion mediate the relationship between an error-avoidance climate and poor leadership. Furthermore, they found that an error-avoidance climate moderates the relationship with moral disengagement, concluding that the relationship between an error-avoidance climate and moral disengagement is stronger when leaders have a high preventative-regulatory focus and a high incentive-regulatory focus. Zhang and Ma examined how employees' expected and perceived trust, a fundamental emotional concept in organizational behavior, influence impression management. They determined that trust promotes socially positive management and reduces selfish behaviors. They also found that higher levels of trust were positively associated with prosocial behaviors; employees with high expected trust but low perceived trust resorted to selfish strategies, while employees with low expected trust but high perceived trust exhibited stronger prosocial tendencies. Furthermore, Wang et al. investigated the relationship between an error management climate, psychological safety, and employee piracy through the moderating role of risk-taking characteristics in organizations. The results show that an error-management environment has a significant positive effect on employees' piracy innovation behaviors; psychological safety plays a mediating role between an error-management environment and piracy innovation behaviors; and risk-taking characteristics play a moderating role in the relationship between psychological safety and employees' piracy innovation behaviors.

Zhang Y. et al. investigated the mediating role of perceived organizational support and organizational identification in increasing job commitment through leader tolerance. They found that leader tolerance significantly increased employees' job commitment and confirmed the mediating roles of perceived organizational support and organizational identification in the relationship with job commitment.

Erden investigated the impact of teachers‘ psychological capital on the quality of work life based on the mediating effect of emotions. The mediating role of emotions in the relationship between teachers' psychological capital and perceptions of quality of work life was fully supported, and he found a significant correlation between their psychological capital, perceptions of quality of work life, and emotions. Psychological capital was found to have a significant and positive impact on both emotions and perceptions of quality of work life.

In several studies featured in this special issue, the significance of digital transformation for organizational innovation is underlined. For instance, Sibassaha et al. investigated the impact of digital transformation on employees' innovative behaviors in Brazil, examining the moderating roles of organizational culture and leadership styles. Their findings emphasize that the ability to cope with challenges and the presence of organizational cultural support play a crucial role in facilitating the influence of digital transformation on innovation. Notably, they also demonstrate that higher levels of transformational leadership do not necessarily enhance innovation in organizations undergoing digital transformation—an observation of considerable theoretical value. Similarly, Li et al. showed that digital leadership is associated with more favorable shifts in the emotional commitment of younger employees. They further argue that employee empowerment and voice behaviors mediate this relationship, creating a chain-mediating mechanism between digital leadership and emotional commitment. Along the same lines, Chen et al. revealed that managerial cognition exerts a significant positive effect on innovation performance; moreover, compositional capability mediates this link, while the updating of organizational routines strengthens the positive association between managerial cognition and compositional capability.

Other contributions have examined organizational innovation through the lenses of leadership and conflict. Wang and Duan, for example, analyzed intergenerational diversity and team innovation by considering the roles of conflict and shared leadership. Their results indicate that generational diversity predicts both cognitive and affective conflict, which exert opposing influences on team innovation. Shared leadership strengthens the positive relationship between cognitive conflict and team innovation, thereby enhancing the indirect effect of intergenerational diversity. However, it was found to have no significant impact on the link between affective conflict and team innovation. In a related study, Liu M. et al. explored how team reflexivity affects employees' feedback-seeking behaviors. They report that shared mental models mitigate the effect of team reflexivity on transactive memory systems (TMS). These findings suggest that recognizing the roles of reflexivity, TMS, and shared mental models can help organizations design more effective strategies to encourage feedback-seeking behaviors among employees.

In the context of hybrid work, Kim and Yoon examined the impact of empowering leadership on adaptive performance in Korea, focusing on the serial mediating effects of knowledge sharing and employee agility. Their results confirm that empowering leadership positively influences adaptive performance and that knowledge sharing and agility partially mediate this relationship.

The special issue also features studies that investigate the interplay between intra-school relations and leadership. Kaya analyzed the mediating effects of professional resilience and job satisfaction in the relationship between transformational leadership and teachers' creativity. The findings reveal that transformational leadership significantly predicts job satisfaction and professional resilience, though it does not have a statistically significant direct effect on creativity. Nonetheless, professional resilience mediates the link between transformational leadership and creativity. Taken together, these results suggest that the prevalence of transformational leadership in schools may enhance both teachers' resilience and their creative potential.

Kandemir examined the mediating role of school effectiveness in the relationship between transformational leadership and workplace exclusion. The study shows that both school effectiveness and transformational leadership exert significant negative effects on workplace exclusion, with school effectiveness mediating this relationship. Practical recommendations include offering in-service training on transformational leadership and school effectiveness for administrators as well as providing teachers with opportunities for professional development and participation in decision-making, thereby helping to reduce workplace exclusion. Similarly, Dere highlighted that the development of STEM intelligence and the acquisition of scientific attitudes positively influence student motivation in higher education. The findings further indicate that female students hold more favorable attitudes toward scientific research. As positive attitudes toward research and researchers increase, so too does motivation for STEM. Improvements across verbal-linguistic, logical-mathematical, spatial, interpersonal, intrapersonal, and naturalistic intelligences were also found to contribute to these positive attitudes, thereby enhancing STEM motivation.

Finally, the literature also reveals conceptual intersections between organizational leadership and women's leadership. Some authors have therefore turned their attention to this dimension. Palomo-Vadillo et al. developed an index to measure gender-focused investment practices within organizations, proposing and validating an instrument designed to capture multiple dimensions of gender-lens investing (GLI) from both academic and practitioner perspectives. Likewise, Eratli Sirin and Öz identified gender-based negative experiences in organizational contexts, noting that female observers are disproportionately subject to the glass ceiling compared with their male counterparts. They recommend the implementation of positive organizational discrimination measures in the selection of football observers to address this imbalance.

## Conclusion

Based on the papers collected here, it can be stated that there is a relationship between leadership and creativity, and it has been scientifically demonstrated that this relationship plays an important role in the development of organizational culture and behavior. Furthermore, it is understood that the topic is related to employee emotions toward organizations; trust, especially, is a key factor in promoting a positive organizational culture. Moreover, an ethical climate also supports innovation, organizational identification, and healing. Nowadays, leadership needs creative thinking and an ethical organizational environment. In addition, digitalization has started to affect organizational leadership and psychology.

Organizational leadership is related to many behavioral and cultural topics such as innovative behavior, creativity, employee outcomes, organizational commitment, team reflexivity, social support, inclusion, sustainability, team building, developmental feedback, motivation, emotional intelligence, work engagement, organizational quality, organizational identification, emotions, psychological safety, social support, life satisfaction, job satisfaction, perceived power, ethical behavior, professional development, school health, and change. When employees experience positive emotions, such as happiness, love, and respect, they are motivated to discard time-tested or automatic (everyday) behavioral scripts in favor of novel, creative, and often unscripted paths of thought and action. Therefore, a leader's ethical behavior that puts employees' needs and wellbeing first, making them feel respected and accepted, will strengthen employee-leader relationships and create a positive organizational climate.

From these study results, we understood that creativity, ethical behavior and atmosphere, emotions, and trust in organizational leaders correlate strongly with each other. Furthermore, based on the results, we can say that leadership, creativity, and ethical behaviors affect school culture and transformational organization behavior in schools. We can also claim that creativity in organizations and schools is important. Moreover, managers and organizational leaders have to focus on developing a creative and innovative organizational culture combined with an ethical atmosphere. Finally, leaders' motivation and dedication inspire team transformation and adaptability. Thus, these leadership competencies are instrumental in sustaining a competitive edge and driving organizational growth amidst a dynamic business environment.
