# Correction: Editorial: Creative organization development through leadership

**DOI:** 10.3389/fpsyg.2025.1720152

**Published:** 2025-11-06

**Authors:** Osman Titrek, Carlos Francisco de Sousa Reis, Pavel Jurs, Gözde Sezen-Gültekin, Pablo Garcia Sempere

**Affiliations:** 1 Faculty of Education, Sakarya University, Sakarya, Türkiye; 2 Faculty of Psychology and Educational Sciences, University of Coimbra, Coimbra, Portugal; 3 Centre of Pedagogy and Social Work, Riga Technical University Liepaja Academy, Liepaja, Latvia; 4 Faculty of Education Sciences, University of Granada, Granada, Spain

**Keywords:** creativity, leadership, emotions, leadership types, organizational behavior

In the published article, a reference was erroneously written in the **References** section.

The original reference appeared as:

“Vural, T., and Bayrakci, Gültekin, S. (2024). “Basics and theories of creativity”, in Creative Learning for Entrepreneurship, eds. Rae, D. Frank, R., Hoare, M. (Octaedro Publishing, Barcelona). Available online at https://octaedro.com/wp-content/uploads/2024/03/9788410054127.pdf”

The correct reference appears below:

“Vural, O. F., Titrek, O., Bayrakci, M., and Sezen Gultekin, G. (2024). “Basics and theories of creativity”, in *Creative Learning for Entrepreneurship*, eds. D. Rae, R. Frank, and M. Hoare (Barcelona: Octaedro Publishing). Available online at: https://octaedro.com/wp-content/uploads/2024/03/9788410054127.pdf (Accessed August 15, 2025).”

Author Gözde Sezen-Gültekin was erroneously spelled as Gozde Sezen-Gultekin.

The original version of this article has been updated.

